# Endotoxin tolerance modulates TREG and TH17 lymphocytes protecting septic mice

**DOI:** 10.18632/oncotarget.26919

**Published:** 2019-05-28

**Authors:** Mariana M.C. Andrade, Suely S.K. Ariga, Denise F. Barbeiro, Hermes V. Barbeiro, Rosangela N. Pimentel, Ricardo C. Petroni, Francisco G. Soriano

**Affiliations:** ^1^ Laboratório de Investigação Médica – LIM 51, Faculdade de Medicina, Universidade de São Paulo (FMUSP), São Paulo, Brazil

**Keywords:** sepsis, lymphocytes, Treg, Th17, tolerance

## Abstract

**Background**: Tolerance induces a regulated immune response to infection. We hypothesized that tolerance induction modulated profile of T regulatory cell (Treg) and T lymphocyte 17 (Th17) cells and is related cytokine released in septic animals. **Methods**: Male black *C57/6 mice* received subcutaneous (s.c.) injections of lipopolysaccharide (LPS) (1 mg/kg) for 5 days, on day 8^th^ was made cecal ligation and puncture (CLP). Blood and spleen tissue were collected for cell analysis and cytokines measurements. **Results**: Cytokines (interleukin 2 (IL-2), interleukin (IL-6), transforming growth factor β (TGF-β) and interferon γ (INF-γ)) related to Treg and Th17 stimulation were elevated in the spleen of tolerant animals compared to sham. Treg and Th17 lymphocytes showed an increased amount in blood (Treg: 920 ± 84 cells vs. 1946 ± 65 cells, sham vs. tolerant; Th17:38321± 1954 cells vs. 43526 ± 7623 cells, sham vs. tolerant) and spleen (Treg: 5947 ± 273 cells vs. 16521 ± 486 cells, sham vs. tolerant; Th17: 26543 ± 2944 cells vs. 64567 ± 5523 cells, sham vs. tolerant). Treg (135±23 cells) and Th17 (1590 ± 256 cells) cells were reduced in blood of septic animals compared to sham, while CLP tolerant animals presented an increasing number of these cells. Lymphocyte Th17IL6+ were elevated in tolerant and CLP tolerant animals in the blood compared to sham. **Conclusion**: LPS tolerance was associated with increasing population of Treg and Th17. LPS tolerance reduces the hyper inflammatory response with immunoregulation exerted by Treg and Th17 cells protecting from septic damage.

## INTRODUCTION

Sepsis has been defined as a dysregulated immune response caused by an infection or bacterial components. Recently, life-threatening organic dysfunction has been added to the definition of sepsis [1, 2]. Lipopolysaccharide (LPS) exerts its effects via Toll-like receptor 4 (TLR4) one of the most potent known inducer of inflammation [3]. During a massive or persistent infection the inflammatory response becomes excessive causing multiple organ dysfunction and progressive development of immunosuppression due to lymphocyte apoptosis occurs late [4–6].

On the other hand, previous induction of LPS tolerance reduces monocyte and lymphocytes apoptosis as well reduces multiple organ failure [7–9]. Tolerance to LPS is characterized by resistance to lethal doses of LPS or CLP [7–9]. The profile of Treg and Th17 cells after LPS tolerance and subsequent sepsis induction has not been studied. Regulatory T cells (Treg) are a subset of T suppressor cells essential for the good maintenance of self-tolerance and immunological homeostasis through the TGFβ and interleukin 10 (IL-10) [10]. Another subset of CD4 lymphocytes are Th17, lymphocyte CD4+ (T CD4+) differentiates into Th17 cells in the presence of IL-6 and TGF-β [11–13]. Th17 cells had been reported to regulate neutrophil proliferation and tissue infiltration [14]. The role of these cells in the immunology of sepsis is still controversial [15–17].

The hypothesis is that the balance of Treg and Th17 cells determined immune regulation observed in LPS tolerance. The aim of this study was to evaluate the profile of lymphocytes after the induction of sepsis by CLP in animals submitted to LPS tolerance.

## RESULTS

### LPS tolerance induce splenic cytokines for Treg and Th17 control

**Figure 1:**
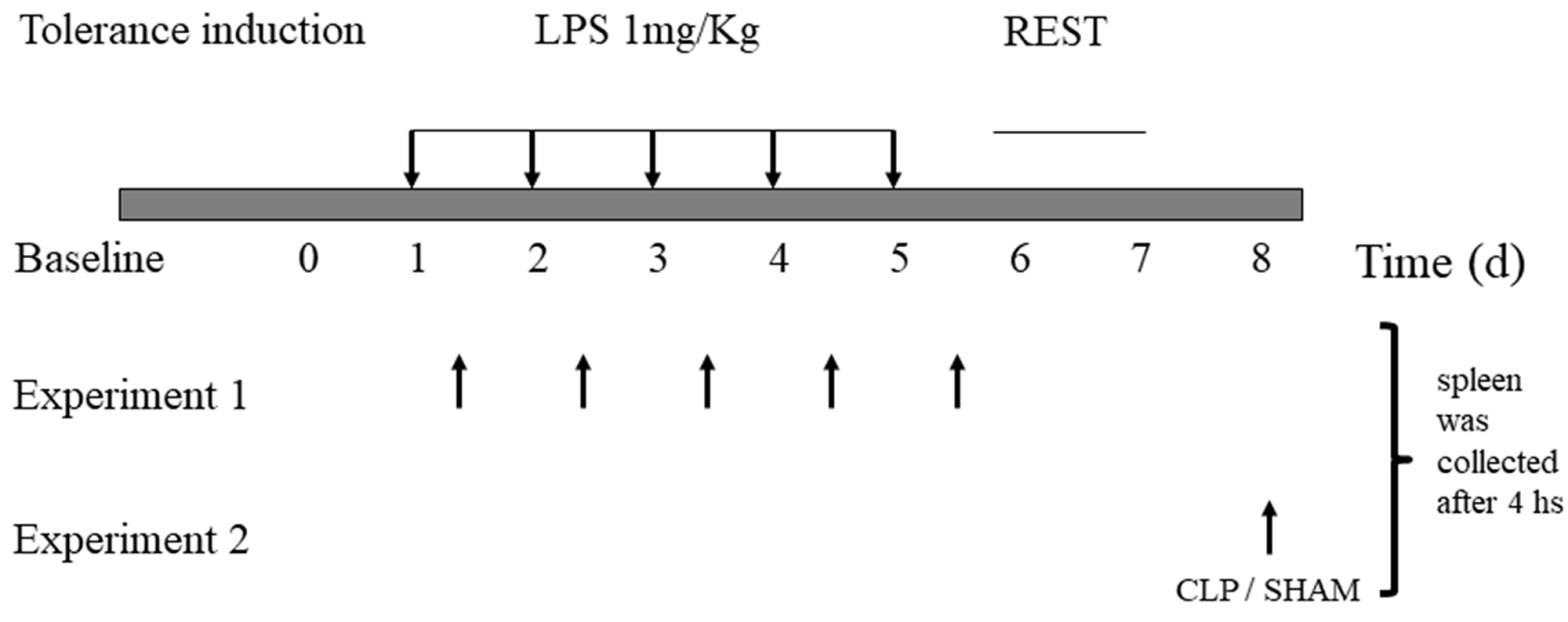
Lipopolysaccharide-induced tolerance in mice. C57/6 mice were treated daily with 1 mg/kg of lipopolysaccharide (LPS; tolerant group, n = 30) or saline placebo (naïve group, n = 30) for 5 d. Experiment 1- spleen were collected from animals sacrifeied every day, 4 houras after LPS induction dose. Experiment 2- On day 8, animals were submitted to CLP or SHAM surgery, and samples were collecte after 4 houras.

**Figure 2:**
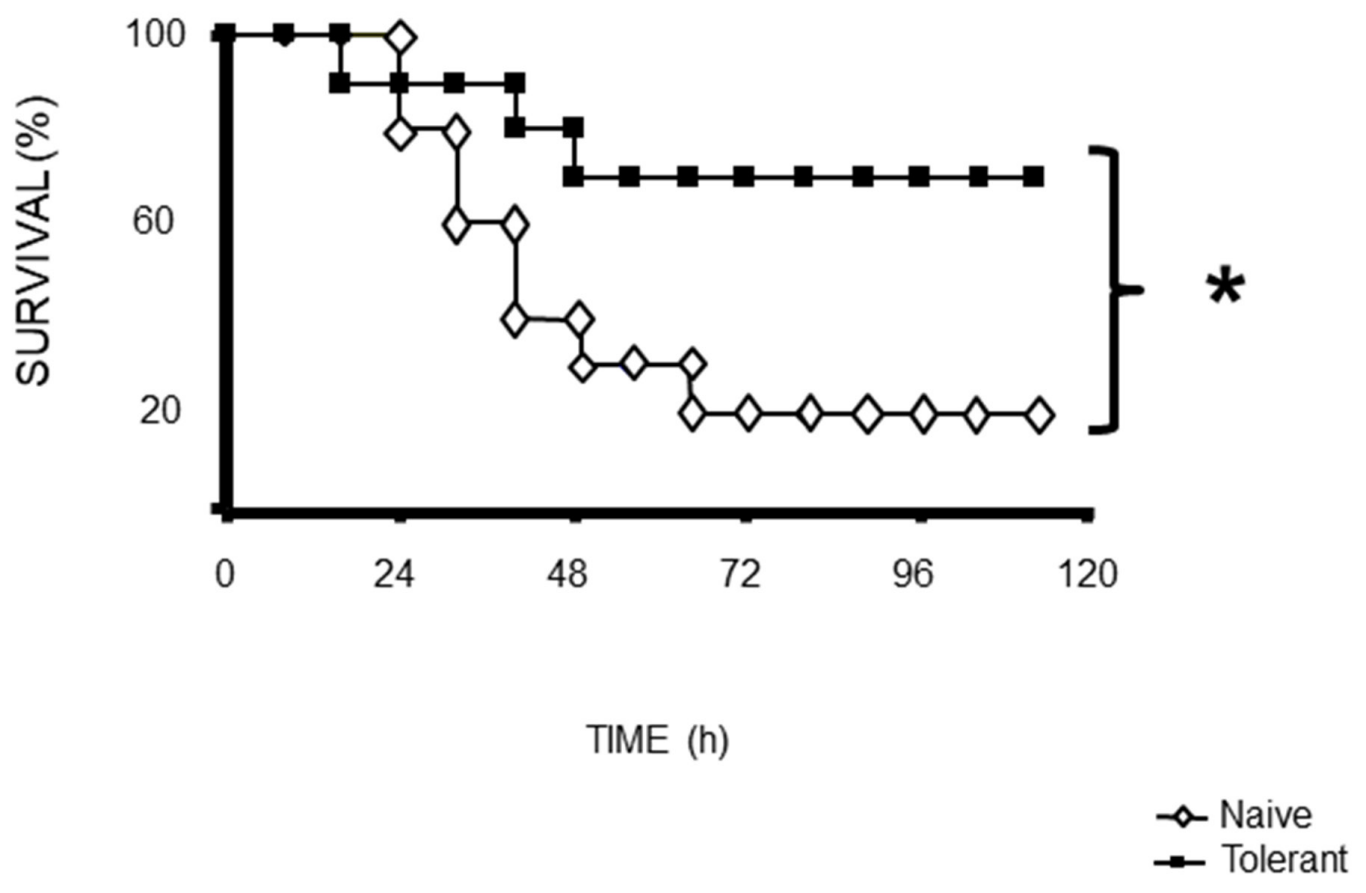
Mortality following CLP (b) was 80% in naïve mice after 72 h, 40% in tolerant mice after 72 h. Data from of 30 animals per group. ^*^p<0.05 for a difference between tolerant and naïve groups.

A schematic representation of the procedure to indeuce tolerance in the animals (Figure 1). Survival was assessed after CLP challenge, and survival was assessed every 8 h up to 72 h after CLP. After 72 h, there was no change in survival. (Figure 2)

**Figure 3:**
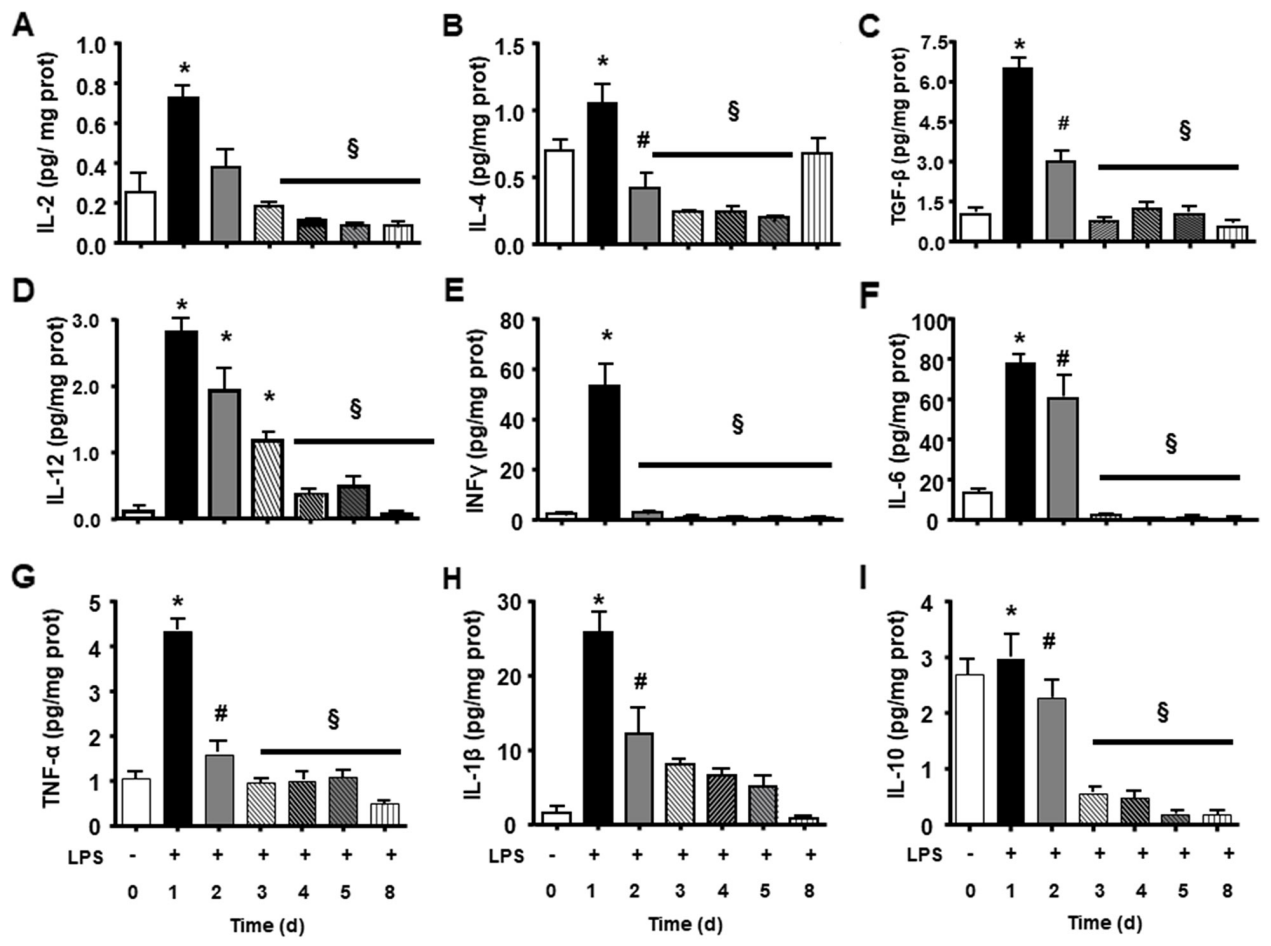
Splenic inflammatory cytokines and growth cytokines during LPS tolerance development. L-2 **(A)**, IL-4 **(B)**, TGF-β **(C)**, IL12 **(D)**, INFγ **(E)**, IL-6 **(F)**, TNFα **(G)**, IL1ß **(H)**, and IL-10 **(I)** interleukin levels were determined in basal group (BASAL 0), and 4 hours after each LPS injection, in days 1, 2, 3, 4, 5 and 8 of tolerance induction. Animals per period n=8, data are presented as mean ± SD. (^*^) p<0.05 compared to BASAL group (n=6 for each group); (§) p<0.05 compared to day 1; (#) p< 0.05 compared to days 3, 4, 5 and 8.

**Figure 4:**
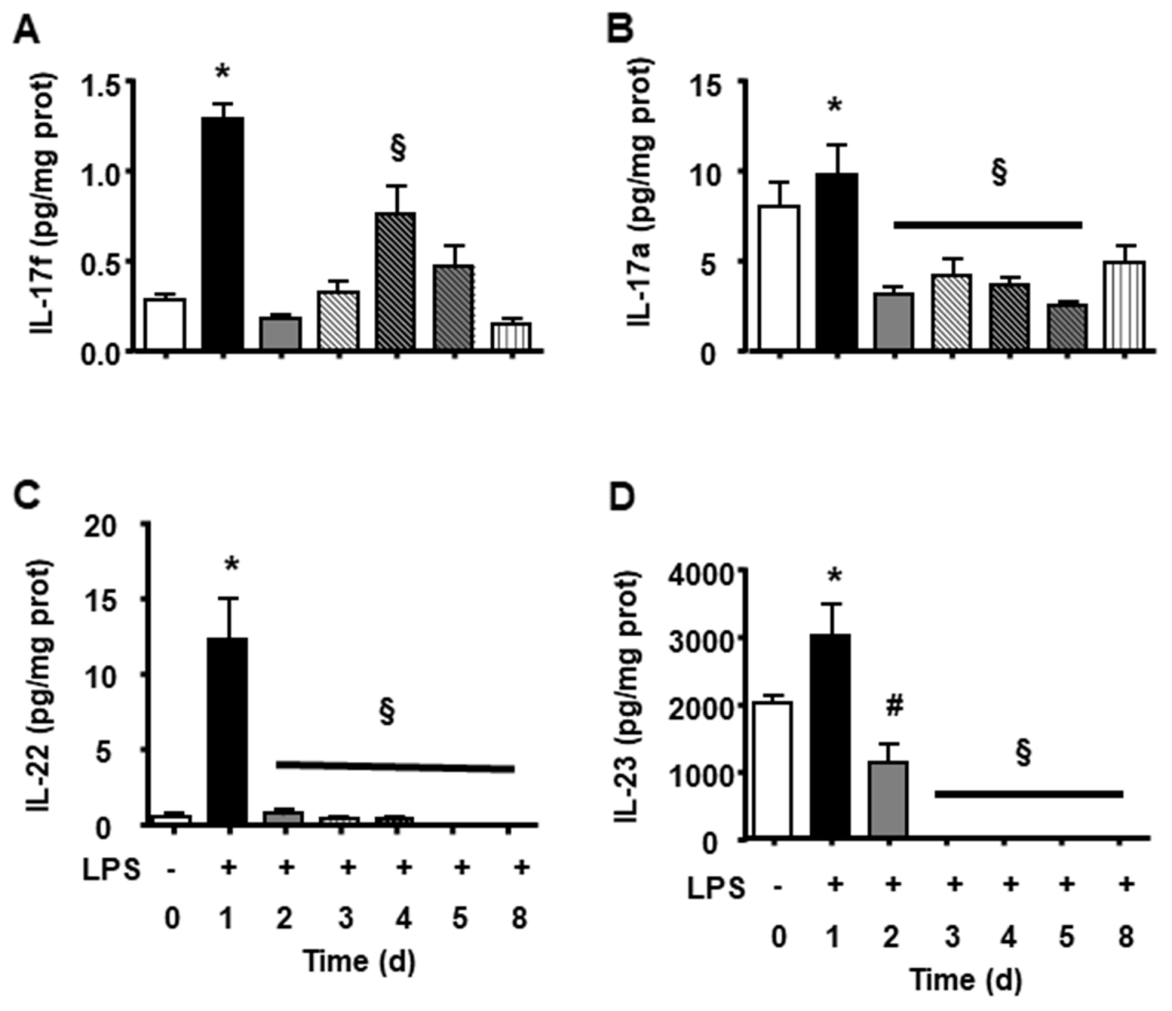
Splenic Th17 cytokines during LPS tolerance development. IL-17f **(A)**, IL-17a **(B)**, IL-22 **(C)** and IL-23 **(D)** interleukin levels were determined in basal group (BASAL), and 4 hours after each LPS injection, in days 1, 2, 3, 4, 5 and 8 of tolerance induction. Animals per period n=8, data are presented as mean ± SD. (^*^) p<0.05 compared to BASAL group (n=8 for each group); (§) p<0.05 compared to day 1; (#) p< 0.05 compared to days 3, 4, 5 and 7.

We observed that endotoxin tolerance induced an elevation of pro-inflammatory cytokines post first doses. The following doses caused a lower cytokine production (Figure 3A, 3D, 3E, 3F, 3G, 3H). Interestingly anti-inflammatory cytokines presented high basal amount, and the first LPS dose caused a lower increase and further doses caused reduction in all measurements (Figure 3B, 3C, 3I). LPS tolerance increased spleen cytokines involved in lymphocyte activation and proliferation, in that way there were a milieu for lymphocyte proliferation. INF-γ and IL-2 cytokines that stimulate lymphocytes, present an intense peak after first doses of LPS and the next doses did not cause any increase. Th17 cytokines present a similar profile as pro-inflammatory cytokines with a high elevation after first LPS injection and a subsequent smaller elevation after each LPS doses (Figure 4A, 4B, 4C and 4D).

### The LPS tolerance effect on regulation of CD4, Treg and Th17 lymphocytes

**Figure 5:**
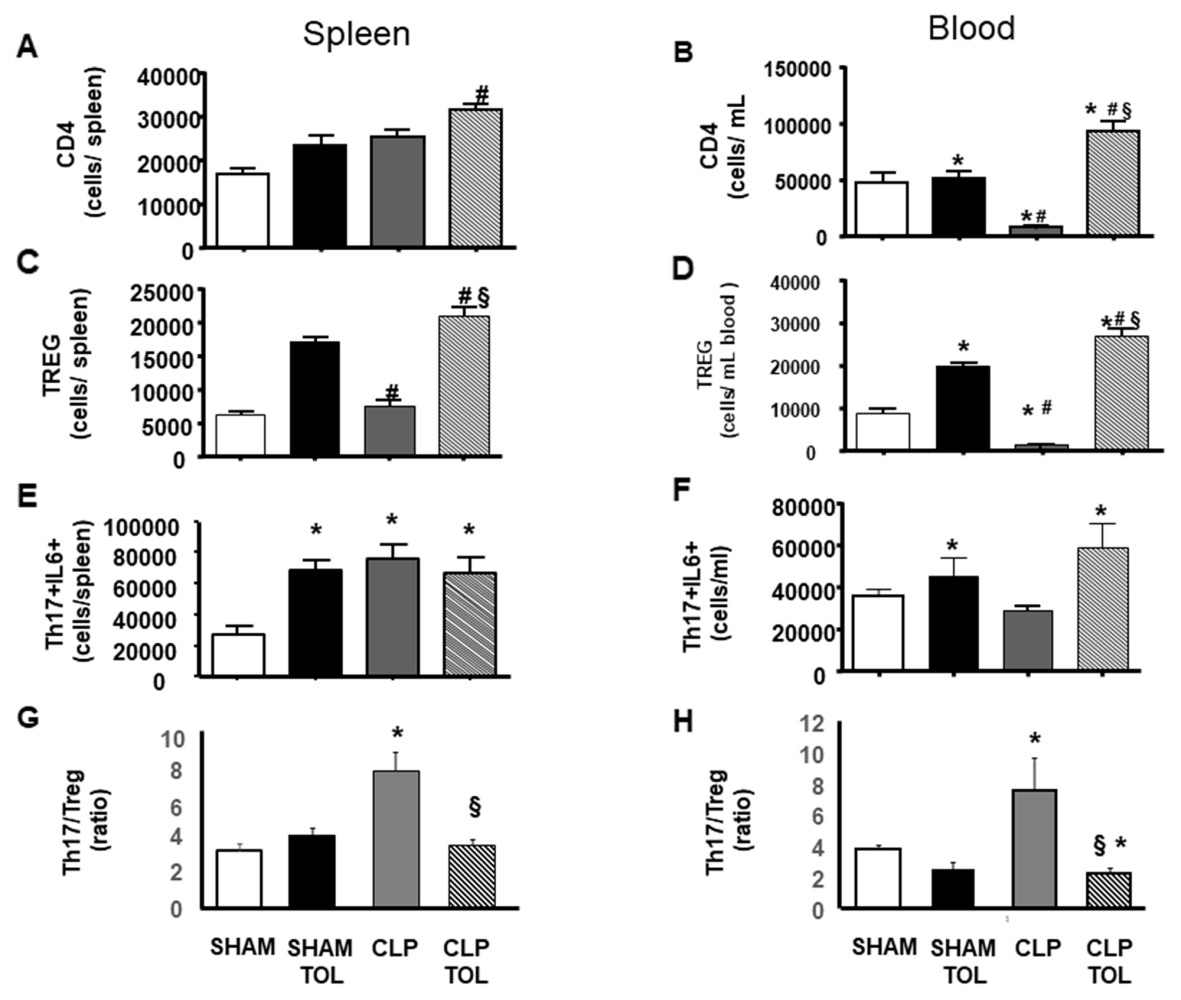
LPS tolerance effect on CD4, Treg and Th17 in septic animals. CD4, Treg and Th17 was quantified in SHAM, SHAM Tolerant, CLP and CLP Tolerant groups. The quantification was performed by flow cytometry in spleen cells and blood cells. CD4 quantification in blood **(A)** and spleen **(B)**; CD4^+^CD25^+^FOXP3^+^ (Treg cells) amount of cells in spleen **(C)** and blood **(D)**. Quantification of CD4+IL17+ IL-6 **(E)** spleen and **(F)** in blood, and Th17-IL6/Treg ratio **(G)** in spleen and **(H)** in blood in. Animals per group n=8, data are presented as mean ± SD. (^*^) p<0.05 compared SHAM group; (#) p<0.05 compared SHAM TOL group; (§) p<0,05 compared to CLP group.

Animals subjected to CLP presented an important decrease in CD4, followed by reduction in Treg and increase in Th17 amount (Figure 5B, 5D, 5F). We observed in the spleen and blood of tolerant animals subjected to CLP an intense increase in population of CD4 (Figure 5A, 5B) and higher increase of Treg (Figure 5C, 5D). The increasing effect was noted in analysis of the LPS tolerance without the challenge of CLP. In addition, tolerant animals subjected to CLP showed a significant increase in the blood and spleen CD4 T lymphocytes (Figure 5A, 5B). In peripheral blood (Figure 5C) and spleen (Figure 5D) regulatory T cells had a significant increase when compared to SHAM, tolerant SHAM and CLP animals.

While we observed a decreased number of Th17 (data not shown) and Th17-IL6 cells in the blood of CLP animals (Figure 5F), tolerant CLP animals presented elevation of Th17 lymphocytes in the blood and spleen (data not shown). Significant increase of Th17 producing IL-6 lymphocytes in the blood of CLP tolerant animals was verified (Figure 5E). In the spleen (Figure 5F), we observed a significant increase of Th17-IL-6 in tolerant SHAM, CLP and CLP tolerant animals when compared to SHAM. The ratio of Th17-IL6 to Treg (Th17/Treg) showed that CLP increases the ratio i.e. there is a more pro inflammatory profile in spleen and blood (Figure 5G and 5H), while tolerant animals preserved the balance of Th17/Treg.

### Splenic cytokines production in LPS tolerant animals after Cecal Ligation and Puncture (CLP)

**Figure 6:**
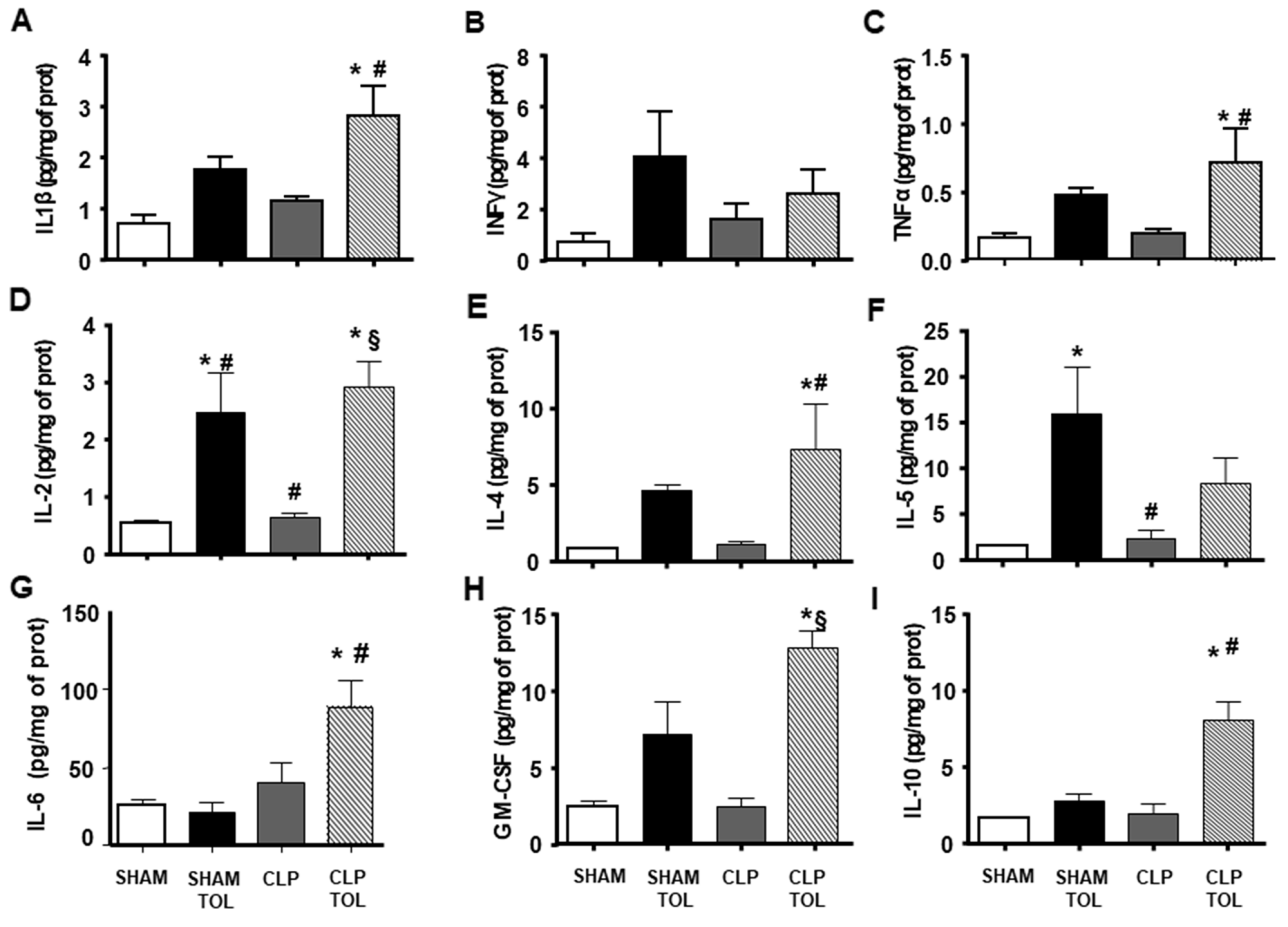
The LPS tolerance effect on splenic pro-, anti-inflammatory and growth cytokines in septic animals. IL1ß **(A)**, INFγ **(B)**, TNFα **(C)**, IL-2 **(D)**, IL-4 **(E)**, IL-5 **(F)**, IL-6 **(G)**, GM-CSF **(H)**, IL-10 **(I)** interleukin levels were determined in SHAM, SHAM Tolerant, CLP and CLP tolerant group. Animals per group n=8, data are presented as mean ± SD. (^*^) p<0.05 compared to SHAM group; (#) p<0.05 compared to SHAM TOL group; (§) p<0,05 compared to CLP group.

Pro and anti–inflammatory cytokines of spleen did not present alteration in CLP and SHAM animals (Figure 6A-6I). Tolerant animals after CLP presented an elevation in all measured cytokines (Figure 6A-6I).

**Figure 7:**
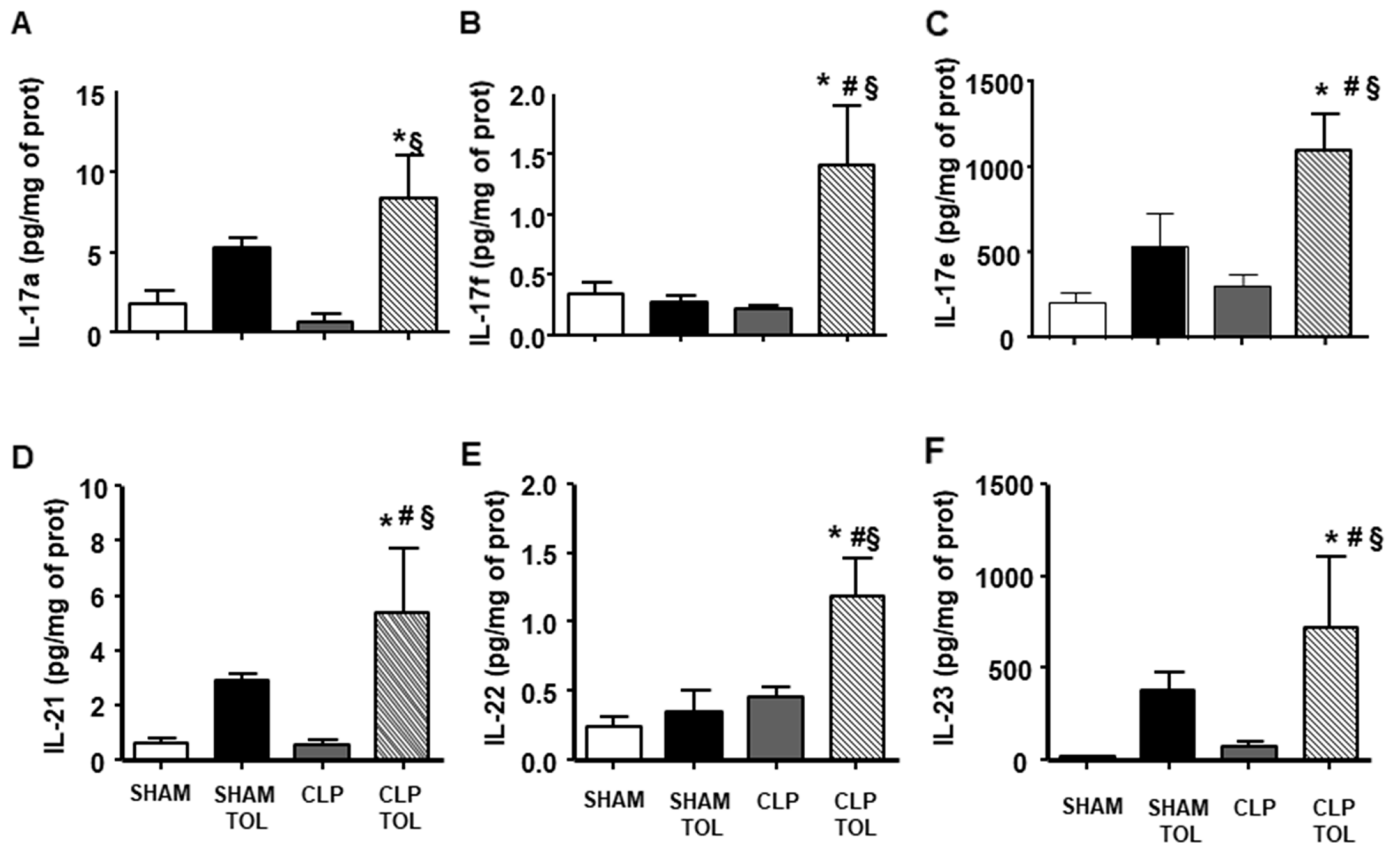
The LPS tolerance effect on splenic Th17 cytokines in septic animals. IL-17a **(A)**, IL-17f **(B)**, IL-17e **(C)**, IL-21 **(D)**, IL-22 **(E)** and IL-23 **(F)** interleukin levels were determined in SHAM, SHAM Tolerant, CLP and CLP tolerant group. Animals per group n=8, data are presented as mean ± SD. (^*^) p<0.05 compared to SHAM group; (#) p<0.05 compared to SHAM TOL group; (§) p<0,05 compared to CLP group.

The cytokines family of Th17 lymphocytes in the spleen did not present alteration after CLP (Figure 7). On the other hand, we observed a significant increase in the levels of these cytokines in CLP tolerant animals. Tolerant Sham group presented higher IL-17a, IL-17e, IL-21, IL-23 (Figure 7A, 7C, 7D, 7F) compared to Sham. Tolerant CLP group presented elevated IL-17F, IL-6 and IL-22 significantly compared to SHAM animals, tolerant SHAM and CLP. Tolerant animals submitted to CLP presented a strong IL-17 cytokines family answer in the spleen tissue.

## DISCUSSION

Our study shows that during the induction of tolerance all cytokines measured increased with the first dose of LPS, presenting a progressive gradual decrease. It is interestingly to note that cytokines IL-2, IL-6 and TGF-β are factors for Treg and Th17 differentiation and proliferation. LPS tolerance presented the capacity to activate and induce proliferation of lymphocytes and maintain activated even after sepsis. The higher amount of T cells in tolerant animals indicates that cell proliferation was sustaining elevated up to the 8^th^ day compared to control. After sepsis induction, tolerant animals persited with a significant higher amount of Treg and Th17 cells and cytokines related to T cell activation (IL- 2). Literature report that a defective T-cell activity and cytokine secretion correlates with increased mortality in sepsis [6] it is interestingly to highlight that LPS tolerance reverted these alterations. We can see that CLP in naïve animals caused an important reduction in the number of Treg in spleen and blood, on the other hand, Th17 was elevated in the spleen and blood. Tolerant animals submitted to sepsis (CLP) were protected of a systemic (blood) reduction of Treg and Th17 compared to naïve submitted to CLP.

Correlated to the amount of Th17 and Th17-IL-6 cells in blood and spleen of septic tolerant animals there were higher production of Th17 cytokines. The IL-17A and IL-17F have proinflammatory properties and act on a broad range of cell types to induce the expression of another proinflammatory cytokines [18, 19]. Th17 cells secrete also IL-21 and IL-23 that have the role of immune cells growth and stabilization factor [20]. In addition, Th17-derived cytokines such as IL-17, INFy, TNF-α and GM-CSF induce the recruitment, activation, and prolonged survival of neutrophils at inflammatory sites increasing bacteria clearance [21]. We have reported previously an increase of neutrophil infiltration at the infectious site in septic tolerant mice [22, 23]. Literature reports that IL-17A receptor blockade correlated with declining levels of proinflammatory cytokines [24] and a decrease in the neutrophils migration to the site of infection and increased bacteremia [25].

On the other hand, tolerant or CLP tolerant animals showed a significant increase in splenic cytokines IL-2, IL-4, INF-γ, IL-5, granulocyte-macrophage colony-stimulating factor (GM-CSF), IL-6 and anti-inflammatory cytokine IL-10 in the spleen. The increased IL-10 and IL-4 anti-inflammatory cytokines expression corroborates to other previous authors, these cytokines contributes to decreased mortality rate in endotoxemia or sepsis [26, 27]. Cells T regulatory act on immune system homeostasis via cytokines IL-10 and TGF-β [10]. Literature data confirmed that IL-10 was an important tool in the modulation of inflammatory response [28]. Therefore, Th17 and Treg not only presented elevated amount but producing more cytokines confirmed also activity of these cells.

Regulatory T cells (Treg) have been shown of central importance for the maintenance of immune homeostasis and self-tolerance [29, 30]. During infection, Treg can prevent excessive pathologies associated with immune response and increased survival [31–33]. Our LPS tolerance data have shown a reduction in mortality and immunopathology. We can hypothesize that the Treg cells found in tolerance are more an immunological aspect of the benefit of tolerance in sepsis. Literature have shown an improved survival rate in experimental sepsis after adoptive transfer of small numbers of ***ex vivo*** activated CD4^+^ CD25^+^ Treg from healthy to septic animals [16, 34]. On the other hand, Treg depletion with anti-CD25 monoclonal antibody (mAb), showed no effect in murine sepsis [15, 17]. In addition, only Treg-competent animals recovered after 36 hours from insult [33, 34]. We have shown in the current study a reduction in mortality more consistent after sepsis in tolerant animals confirming the benefit of Treg cells induced by tolerance [8, 9, 34]. Nascimento et al. evaluating septic mice verified an increase number of Treg cells in survivor’s animals confirming the importance of these cells for sepsis recovery [35]. Taking together with our study, the CLP tolerant animals presented elevated amount of Treg cells in blood and spleen, reducing mortality from a first septic event [8, 9, 36].

### The imbalance of Treg and Th17 during sepsis

The CLP-induced polymicrobial sepsis model has contributed to our knowledge of the involvement of immune components in sepsis disease. Previous experimental studies for sepsis have focused on attenuating the inflammatory response and have ignored the progressive development of immunosuppression [34, 37]. Therefore, the therapy should block the inflammatory overactivation and keep clearance of the pathogen.

The role of Foxp3+ regulatory T (Treg) cells is ambiguous in the course of the early hyper-inflammatory and subsequent hypo-inflammatory phases of sepsis. Authors used DEREG (DEpletion of REGulatory T cells) mice model in order to evaluate the role of Foxp3+ Treg cells in the early and late phases of sepsis. There was an increase of Foxp3+ Treg cells to all CD4+ T cells during murine sepsis. DEREG mice depleted of Foxp3+ Treg cells exhibit higher inflammation and mortality rates in early-phase sepsis [34, 37]. This data indicates that Foxp3+ Treg cells limit the hyper-inflammatory response and accelerate recovery. On the other hand, the data do not support a significant role of Treg cells in immune paralysis during late-phase sepsis.

Some authors have associated the increased survival of septic patients with increased amount on blood of Treg and Th17 [38]. We verified in accordance with literature an increased number of Th17 - IL-6 cells and Treg in the blood and spleens from CLP tolerant animals. This data emphasizes that the development of tolerance leads the immune system to the best response to a septic event. However, increasing the amount of Th17 and Treg cells can occur with an unbalance in favor of Th17. The literature reported that the Th17/Treg ratio is directly related to the SOFA score, higher Th17/Treg values presented a high SOFA score correlating with organ damage and mortality [39]. CLP in naïve animals induced an increased ratio of Th17/Treg, that causes imbalance to proinflammatory profile. Again, induction of tolerance preserved the adequate immune balance for the response to sepsis. Taken together the data we can state that tolerance induction was able to produce a regulated immune response after sepsis challenge.

The cecum is essential for the development of the gut associated lymphoid tissue (GALT). Initially independent development of follicle centres occurs, but presence of the commensal intestinal flora is required for diversification of the primary antibody repertoire [40]. An abundance of immunoglobulin (Ig)A- or IgG-producing plasma cells is found in the lamina propria next to macrophages. In the submucosa CD40-CD40L interaction with T cells, centrocytes can also differentiate into plasmablasts or memory B cells. Paneth cells are found at the bottom of these crypts, with the production of anti-microbial peptides [40]. Intestinal DCs expressing aEb7 are believed to stimulate differentiation of forkhead box protein 3 (FoxP3)1 Treg cells after encountering antigens of bacteria. The biofilm in the appendix is thought to act as a ‘safe house’ for commensal bacteria and to facilitate their reinoculation of the gut after a gastrointestinal infection [40].

## CONCLUSIONS

This study demonstrated that the mortality reduction of animals subjected to CLP after the tolerance, is associated with increased production of growth factor cytokines in the period of LPS tolerance induction for Treg and Th17 with consequent increase of Treg cells. Tolerant animals presented a lower value of Th17/Treg after sepsis, that resents a profile less inflammatory.

## MATERIALS AND METHODS

### Animals and LPS tolerance induction

Male *C57/6 mice*, weighing 25 g, 8 weeks old, were used in this study. All animals were treated according to institutional rules for laboratory animal care. All procedures were performed in accordance to the Guide for the Care and Use of Laboratory Animals published by the National Institutes of Health of the USA and EU Directive 2010/63/EU following ARRIVE guidelines. The study protocol was approved by the Research Ethics Committee of the São Paulo School of Medicine (#248#/12).

### LPS tolerance induction

Mice were randomized into 2 groups: the naïve group received subcutaneous (s.c.) injections of 0.2 ml saline, while the LPS-tolerant group received one dose of LPS (1 mg/kg, s.c.) injection each day during 5 days (Escherichia coli 026: B6 Sigma). After the period of 5 days, a rest of two days without manipulation the mice and on the 8th day the animals were euthanized.

### Groups for the study of tolerance development

For the study of cytokines profile during LPS tolerance development there were BASAL animal without any tolerance or operation procedure; and Tolerance development after LPS in every day after 4 hours of LPS. Six animals for each time point in every group. The animals were provided by the animal facility of our institution and were specific pathogen free (SPF). Animals were kept in an acclimatized facility with an automated 12-h dark/12-h light cycle. Food and water were available *ad libitum*.

### Cecal ligation and puncture

Mice were anesthetized with a mixture of ketamine (80 mg/kg) and xylazine (10 mg/kg) given intraperitoneal (i.p.). Cecal ligature and puncture was induced as previously described [23]. Briefly, under aseptic conditions, a 2 cm midline laparotomy was performed to allow exposure of the cecum with adjoining intestine. The cecum was tightly ligated with a 3.0 silk suture at its base, below the ileocecal valve, and was perforated twice with an 18-gauge needle. The cecum was returned to the peritoneal cavity and the laparotomy was closed with 4.0 silk sutures. Animals were returned to their cages with free access to food and water.

### Animal groups

For the study of tolerance and sepsis in the 8^th^ day mice were submitted to CLP or sham surgery. The groups were separated as: SHAM – animal without tolerance with sham operation procedure; SHAM TOL – tolerant animal submitted to SHAM procedure; CLP – animals naïve submitted to CLP operation; CLP TOL – tolerant animals submitted to CLP operation. Ten animals for groups were used, for blood and tissue samples.

### Survival curve

Survival was assessed after CLP challenge. Each group comprised 30 animals, and survival was assessed every 8 h up to 72 h after LPS or CLP. After 72 h, there was no change in survival. (Figure 2)

### Sample collection

#### Blood and splenic cells for flow cytometry measurement

After CLP procedure mice were anesthetized with ketamine (80 mg/kg) and xylazine (10 mg/kg) i.p. Blood samples were obtained by cardiac puncture and centrifuged 400g for 10 minutes. The plasma was removed and the packed red blood was incubated with red cell lysis buffer (0.16M NH_4_CL and 0.17M TRIS). Cells were washed, counted and 10^6^ cells were suspended in PBS. To analyze the splenic cells, the whole organ was collected, macerated, suspended in 2mL of PBS, washed, and incubated in lysis buffer. After that, cells were washed 3 times, counted and suspended 10^6^ cells in phosphate buffer saline (PBS).

#### Determination of cytokine concentrations in spleen

Panels of 13 cytokines were measured using Miliplex® technology (Merck Millipore, Darmstadt, Germany) a multiplex method for cytokine analysis. The panel included: TNFα, INFγ, TGF-β, IL-1β, IL-2, IL-4, IL-10, IL-6, GM-CSF, IL-17A, IL-17E, IL-17F, IL-21, IL-22 and IL-23. The values were normalized by protein content from spleen.

### Splenic proteins for cytokines measurement

Frozen tissues (100mg) were pulverized in liquid nitrogen. Samples were then homogenized in TritonX-100 150 mM NaCl, 10 mM Tris HCl (pH 7.5), 1 % NP40, 1 % sodium oxalate, 0.1 % SDS, and proteolytic enzyme inhibitors (40 μg/mL PMSF 1mM; Sigma, St, Louis, MO). After centrifugation for 40min at 10,000 rpm, the supernatants were collected, and protein concentration was determined by the BCA method (Pierce). Samples were stored at −80 °C.

### Flow cytometry

Flow cytometry analysis was performed using a Guava 8HT (EMD Millipore Corporation, Billerica, MA USA). Splenic and blood cells from control and tolerant mice were collected and labeled with antimouse antibodies (BioLegend, San Diego, CA) for cell surface markers: CD4-PE and CD25- APC/Cy7. After cell membrane permeabilization with saponin, cells were intracellular labeled with antimouse antibodies: FOXP3-PE/Cy7, IL-17a- APC and IL-6-FITC (BioLegend).

### Statistical analysis

Data were expressed as mean ± standard deviation (SD) and analyzed with analysis of variance (ANOVA; a mixed-model, factorial ANOVA). Turkey Test was used to evaluate significant differences between groups. A p value of 0.05 or less was considered to indicate statistical significance.

## Abbreviations

Treg: T regulatory cell
Th17: T lymphocyte 17
LPS: lipopolysaccharide
CLP: cecal ligation and puncture
IL-2: interleukin 2
IL-6: interleukin
TGF-β: transforming growth factor β
INF-γ: interferon γ
TLR4: Toll-like receptor 4
IL-10: interleukin 10
T CD4+: lymphocyte CD4+
IL-21: interleukin 21
IL-23: interleukin 23
IL-6: interleukin 6
IL-17: interleukin 17
GM-CSF: granulocyte-macrophage colony-stimulating factor
mAbs: monoclonal antibody
SIRS: systemic inflammatory response syndrome
SPF: specific pathogen free
PBS: phosphate buffer saline
